# Misophonia: Phenomenology, comorbidity and demographics in a large sample

**DOI:** 10.1371/journal.pone.0231390

**Published:** 2020-04-15

**Authors:** Inge Jager, Pelle de Koning, Tim Bost, Damiaan Denys, Nienke Vulink

**Affiliations:** 1 Department of Psychiatry, Amsterdam UMC (location AMC), Amsterdam, The Netherlands; 2 Department of Otorhinolaryngology, Clinical and Experimental Audiology, Amsterdam Public Health, Amsterdam UMC (location AMC), University of Amsterdam, Amsterdam, The Netherlands; Medical University of Vienna, AUSTRIA

## Abstract

**Objective:**

Analyze a large sample with detailed clinical data of misophonia subjects in order to determine the psychiatric, somatic and psychological nature of the condition.

**Methods:**

This observational study of 779 subjects with suspected misophonia was conducted from January 2013 to May 2017 at the outpatient-clinic of the Amsterdam University Medical Centers, location AMC, the Netherlands. We examined DSM-IV diagnoses, results of somatic examination (general screening and hearing tests), and 17 psychological questionnaires (e.g., SCL-90-R, WHOQoL).

**Results:**

The diagnosis of misophonia was confirmed in 575 of 779 referred subjects (74%). In the sample of misophonia subjects (mean age, 34.17 [*SD* = 12.22] years; 399 women [69%]), 148 (26%) subjects had comorbid traits of obsessive-compulsive personality disorder, 58 (10%) mood disorders, 31 (5%) attention-deficit (hyperactivity) disorder, and 14 (3%) autism spectrum conditions. Two percent reported tinnitus and 1% hyperacusis. In a random subgroup of 109 subjects we performed audiometry, and found unilateral hearing loss in 3 of them (3%). Clinical neurological examination and additional blood test showed no abnormalities. Psychological tests revealed perfectionism (97% CPQ>25) and neuroticism (stanine 7 NEO-PI-R). Quality of life was heavily impaired and associated with misophonia severity (r*s* (184) = -.34 *p* = < .001, *p* = < .001).

**Limitations:**

This was a single site study, leading to possible selection–and confirmation bias, since AMC-criteria were used.

**Conclusions:**

This study with 575 subjects is the largest misophonia sample ever described. Based on these results we propose a set of revised criteria useful to diagnose misophonia as a psychiatric disorder.

## Introduction

Misophonia is a recently recognized condition, characterized by an impulsive aversive physical reaction of irritation, anger, or disgust when confronted with specific, repetitive stimuli (for instance, eating sounds). The word was first used in audiology literature as a hatred of sounds[1]. In 2013 our research group at the Amsterdam University Medical Centers (Amsterdam UMC, location AMC) proposed the first diagnostic criteria for misophonia as a psychiatric disorder[2] (Table 1). Thereafter, research on misophonia has increased vastly. The Amsterdam viewpoint is misophonia is definitely a psychiatric disorder, though there’s no agreement among different research teams. For a recent descriptive overview, we refer to Taylor[3] or Brout et al.[4].

**Table 1:** AMC 2013 diagnostic criteria for misophonia.

AMC 2013 criteria for misophonia
A. The presence or anticipation of a specific sound, produced by a human being (e.g. eating sounds, breathing sounds), provokes an impulsive aversive physical reaction which starts with irritation or disgust that instantaneously becomes anger.
B. This anger initiates a profound sense of loss of self-control with rare but potentially aggressive outbursts.
C. The person recognizes that the anger or disgust is excessive, unreasonable, or out of proportion to the circumstances or the provoking stressor.
D. The individual tends to avoid the misophonic situation, or if he/she does not avoid it, endures encounters with the misophonic sound situation with intense discomfort, anger or disgust.
E. The individual’s anger, disgust or avoidance causes significant distress (i.e. it bothers the person that he or she has the anger or disgust) or significant interference in the person’s day-to-day life. For example, the anger or disgust may make it difficult for the person to perform important tasks at work, meet new friends, attend classes, or interact with others.
F. The person’s anger, disgust, and avoidance are not better explained by another disorder, such as obsessive-compulsive disorder (e.g. disgust in someone with an obsession about contamination) or post-traumatic stress disorder (e.g. avoidance of stimuli associated with a trauma related to threatened death, serious injury or threat to the physical integrity of self or others).

Currently, a total of 797 misophonia subjects has been described in 26 clinical research papers, including five sample studies [2,5,6,7,8]. Only subjects included in the AMC sample[2] and a sample study published last year (Erfanian, Kartsonaki & Keshavarz[9]) had a systematic medical and psychiatric examination. All other samples (of the papers included in our 2018 search) merely used questionnaires to diagnose misophonia.

Without a systematic clinical interview, which is missing in almost three quarters of all described subjects, misophonia symptoms could possibly be better explained by another disorder or results could be influenced by self-report biases (references in S1 Table and S1 Fig).

Therefore, we assessed a new sample of subjects with misophonia symptoms who were referred to the AMC by their general practitioner, which is both quantitatively and qualitatively superior to previous research. The first aim of this study was to determine whether referred subjects with misophonia-like symptoms actually suffered from misophonia using a psychiatric interview conducted by three experienced psychiatrists. The second aim was to determine phenomenology, comorbidity, and demographics of the misophonia sample to address three major issues: 1) whether misophonia should be approached from an audiological or psychiatric/psychological perspective; 2) whether specific psychological profiles, which have been associated with misophonia, such as disgust sensitivity[10], autism-like traits[11,12] and perfectionism[2] are still valid; and 3) whether misophonia is a distinct psychiatric disorder for which diagnostic criteria should be determined.

## Methods

### Subjects

In this sample study, we analyzed data collected from subjects who were referred with misophonia symptoms from 2013 through 2017 at the Department of Psychiatry at Amsterdam University Medical Center (Amsterdam UMC), the Netherlands. This study has been approved by the ethics committee of Amsterdam UMC and the need for informed consent was waived.

Of the 779 examined subjects, 575 subjects met criteria for misophonia. The 204 subjects excluded from this sample were: subjects with primary autism spectrum conditions (ASC), primary attention-deficit (hyperactivity) disorder (AD(H)D), a primary diagnosis on Axis II (varying from schizotypal personality disorder to obsessive compulsive personality disorder) and subjects without a DSM-IV diagnosis. Hearing impairments or audiologic disorders were no exclusion criterion.

### Diagnostic procedures

Assessment of current Axis I and Axis II disorders based on the DSM-IV criteria[13] was determined with the MINI-International Neuropsychiatric Interview Plus[14] (MINI-plus) and sections of the Structured Clinical Interview for DSM-IV Axis II Personality Disorders[15] (SCID II). Based on information obtained from clinical interview, questionnaires or psychiatric history specific sections of the SCID-II relevant to each subject were selected and conducted. DSM-5 was not in use for clinical purposes at our department until 2018. Three psychiatrists, specialized in anxiety disorders and obsessive-compulsive and related disorders, carried out the clinical (medical and psychiatric) interviews.

Somatic assessment consisted of a general physical and neurological examination and a general blood screening. Audiometry was performed with the Hughson-Westlake procedure[16] to obtain hearing thresholds in a random selection of participants (n = 109) in the first 300 subjects. Patients were randomly assigned to three psychiatrists. In a period of 20 months the assessment of one psychiatrist was extended with audiometry. Because the results were clear, we stopped performing audiometry in order not to unnecessarily burden subjects. Air conduction thresholds were measured at all octave frequencies from 0.25 to 8 kHz and bone conduction thresholds were measured at 0.25, 0.5, 1, and, 2 kHz, with adequate masking if necessary. The Pure Tone Average (PTA) was obtained by averaging air conduction thresholds 0.5, 1, 2, and, 4 kHz and hearing loss classification was defined according to WHO-classification[17].

Finally, a variety of self-report questionnaires examined the nature and severity of misophonia symptoms, quality of life, anxiety and depressive symptoms, and personality profile of the subjects. Given the naturalistic nature of the sample, the standard battery of questionnaires at our psychiatry outpatient clinic was used (seven questionnaires), with several additional questionnaires to understand the phenomenology of misophonia and the relation with possible correlated constructs. All questionnaires were administered during intake procedure. A random selection of subjects (n = 56) completed an additional personality questionnaire (see S2 Table). During 4 months all intakes (60 subjects in total) were approached for this additional personality questionnaire, which was completed by 56 subjects.

### Questionnaires

The following questionnaires were administered: *Misophonia Screening List (*see S1 Appendix), *Misophonia Sound List* (MSL; see S2 Appendix), A*msterdam Misophonia Scale*[2] (A-MISO-S), *AMISOS Revised* (AMISOS-R; see S3 Appendix), *Hamilton Depression Rating Scale*[18, 19] (HDRS), *Hamilton Anxiety Scale*[20,21] (HAS), *Symptom Checklist 90 Revised* [22, 23] (SCL-90-R), *Manchester Short Assessment of Quality of life*[24, 25] (MANSA), *Sheehan Disability Scale*[26] (SDS), *WHO Quality of Life-BREF*[27, 28] (WHOQoL-BREF), *NEO-Personality Inventory-revised*[29, 30] (NEO-PI-R), *Autism Spectrum Quotient*[31, 32] (AQ), *Inventory of Interpersonal Situations*[33] (IIS), *Clinical Perfectionism Questionnaire*[34] (CPQ), *Frost Multidimensional Perfectionism Scale*[35] (FMPS), *Disgust Propensity and Sensitivity Scale Revised*[36, 37] (DPSS-R), *Disgust Scale Revised*[38, 39] (DS-R). For more information, see S2 Table.

### Statistical analysis

All statistical analyses were conducted with SPSS statistical package version 24. We report the sample descriptively in terms of means and standard deviations or percentage of the sample, where appropriate. We used independent-samples t-tests to explore whether males and females differed in age of onset and symptom severity (i.e. A-MISO-S or AMISOS-R score). We used multiple linear regression to explore whether certain features were associated with symptom severity. AMISOS-R scores were included as independent variables, and CPQ, FMPS, AQ, DS-R and DPSS-R were included as dependent variables. We confirmed normality of residuals by checking the QQ plot of the model. We treated the full Likert scales as numerical, since assumptions of linear regression were met (residuals were normally distributed) and results are much easier to interpret. Finally, a non-parametric correlation (Spearman’s rho) was calculated to determine whether misophonia symptoms (A-MISO-S) correlated with quality of life (MANSA). No missing scores were imputed and no outliers were removed. We considered P < 0.05 to be statistically significant.

## Results

### Demographics

Our sample was predominantly Caucasian, 69% were female, and 64% had a relationship. Over 85% were employed or studying and 5% were on sick-leave. Mean age at admission was 34.17 years (*SD* = 12.22) and mean age of onset was 13.17 years (*SD* = 7.37). Onset in females was not significantly earlier than in males (*p* = .076). Most subjects (93%) reported a gradual onset of the misophonia symptoms. Clinical interviews with older subjects indicated a chronic course and 33% reported a positive family history of misophonia.

### Comorbidity

The comorbid DSM-IV disorders are shown in Table 2. 72% of subjects diagnosed with misophonia had no comorbid Axis I psychiatric disorder, 22% had one comorbid disorder, and 6% had two or more comorbid disorders. Common comorbid disorders were major depressive disorder (6.8%) and obsessive-compulsive disorder (2.8%). Another 5% of the sample had comorbid AD(H)D and 3% was diagnosed with a comorbid ASC.

**Table 2:** Percentage and absolute frequencies of current DSM-IV Axis I & Axis II disorders in misophonia subjects.

Comorbidity DSM-IV Axis I	% (N)	Comorbidity DSM-IV Axis II	% (N)
**No comorbid diagnosis on Axis I**	**71.8 (413)**	**No comorbid diagnosis on Axis II**	**58.6 (337)**
**Mood disorders**	**10.1 (58)**	**Personality disorder**	**5.0 (29)**
Major depressive disorder	6.8 (39)	Obsessive-compulsive	2.4 (14)
Dysthymic disorder	1.7 (10)	Borderline	1.7 (10)
Bipolar II disorder	0.7 (4)	Avoidant	0.5 (3)
Bipolar I disorder	0.5 (3)	Dependent	0.2 (1)
Depressive disorder NOS	0.3 (2)	Antisocial	0.2 (1)
**Anxiety disorders**	**9.0 (52)**	**Personality traits**	**27.1 (156)**
Obsessive compulsive disorder	2.8 (16)	Obsessive-compulsive	23.8 (137)
Posttraumatic stress-disorder	1.7 (10)	Avoidant	1.4 (8)
Social phobia	1.2 (7)	Borderline	1.2 (7)
Generalized anxiety disorder	1.0 (6)	Narcissistic	0.2 (1)
Specific phobia	1.0 (6)	Antisocial	0.2 (1)
Panic disorder with agoraphobia	0.9 (5)	Schizoid	0.2 (1)
Separation anxiety disorder	0.2 (1)	Schizotypal	0.2 (1)
Anxiety disorder NOS	0.2 (1)	**Mixed personality traits**	**2.6 (15)**
**Autism spectrum conditions**	**2.4 (14)**	Obsessive-compulsive and avoidant	1.4 (8)
Autistic disorder	1.2 (7)	Obsessive-compulsive and borderline	0.3 (2)
Pervasive developmental disorder NOS	1.2 (7)	Avoidant and dependent	0.3 (2)
**Somatoform disorders**	**1.4 (8)**	Obsessive-compulsive and schizotypal	0.2 (1)
Hypochondriasis/BDD	0.9 (5)	Avoidant and narcissistic	0.2 (1)
Undifferentiated somatoform disorder	0.5 (3)	Avoidant and schizoid	0.2 (1)
**Substance related disorders**	**1.6 (9)**	**Diagnosis deferred on Axis II**	**6.6 (38)**
Alcohol dependence	0.7 (4)	**Total**	**100 (575)**
Cannabis or dependence on sedatives	0.5 (3)
Abuse of alcohol	0.3 (2)
**Impulse control disorders**	**2.1 (12)**
Trichotillomania or Excoriation disorder	1.9 (11)
Intermittent explosive disorder	0.2 (1)
**Tic disorders**	**1.6 (9)**
Tic disorder NOS	0.5 (3)
Chronic motor or vocal tic disorder	0.5 (3)
Gilles de la Tourette	0.3 (2)
Tic disorder	0.2 (1)
**Attention Deficit (Hyperactivity) Disorders**	**5.4 (31)**
Attention Deficit Disorder	3.3 (19)
Attention Deficit Hyperactivity Disorder	1.7 (10)
Attention Deficit Hyperactivity Disorder combined type	0.3 (2)
**Other disorders**	**1.4 (8)**
Eating disorder NOS	0.7 (4)
Neurocognitive disorder	0.3 (2)
Schizophrenia	0.2 (1)
Stuttering	0.2 (1)
**Total**	**106.7 (614)**

The majority (59%) had no comorbidity on Axis II. Most prevalent were OCPD (2.4%) and borderline personality disorder (BPD, 1.7%). Obsessive-compulsive personality **traits** were found in 26%. Subjects exhibited especially high morality and clinical perfectionism.

### Misophonia triggers

**Fig 1:**
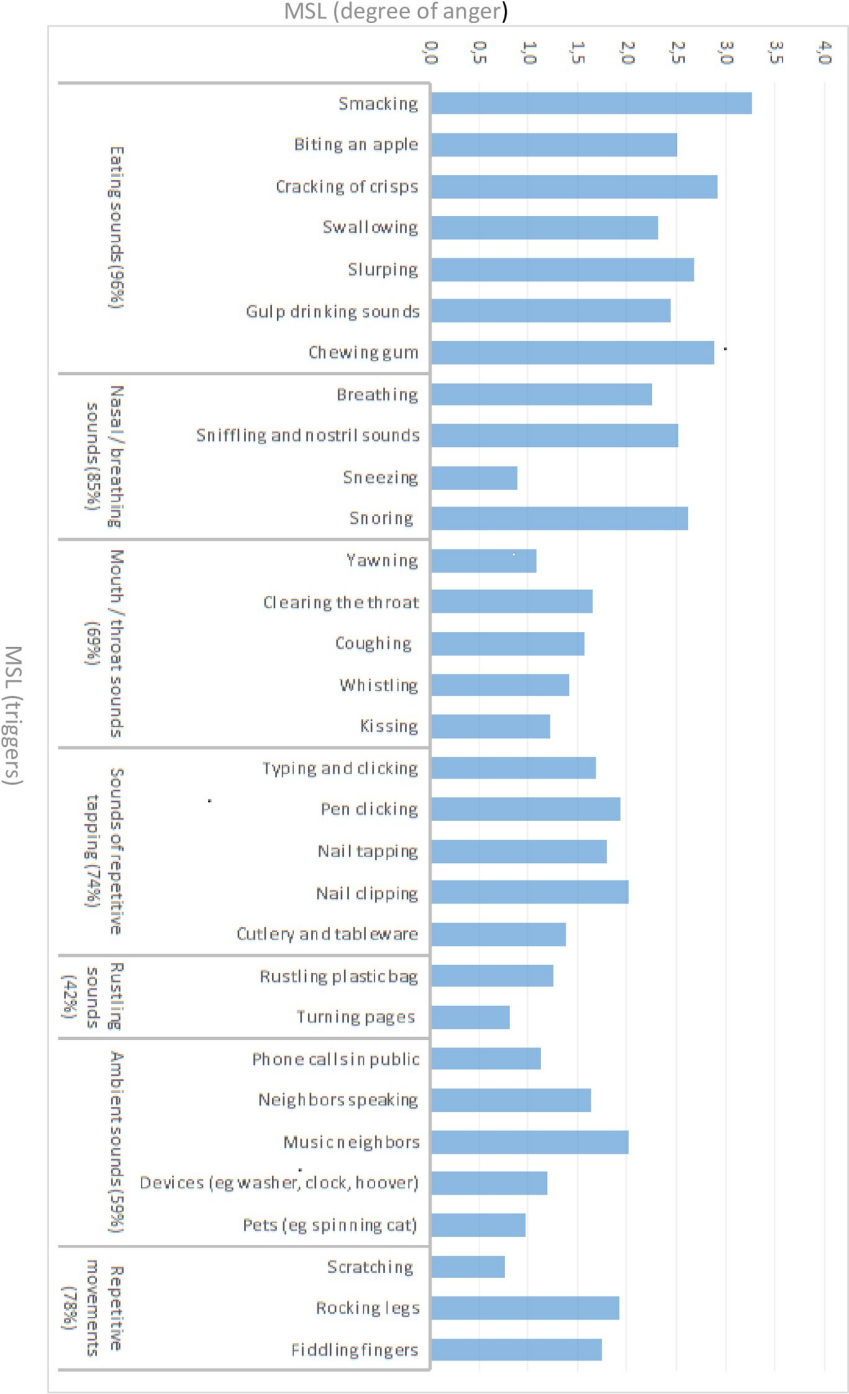
Triggers and provoked anger for misophonia subjects.

Almost all subjects reported to be triggered by eating sounds (96%) followed by nasal and breathing sounds (85%). Subjects were also regularly disturbed by sounds of repetitive tapping or mouth/throat sounds. All triggers are shown in Fig 1.

Visual triggers were often reported, e.g., repetitive movements (68%). We found visual triggers were often directly associated with auditory triggers (e.g., grinding teeth) in clinical interviews. When visual triggers were reported, they occurred secondary to auditory triggers and had less impact than the auditory triggers. When visual and auditory stimuli occurred simultaneously (e.g., hearing and seeing someone chewing gum), subjects reported a more intense response. 59% were bothered by ambient sounds, particularly by sounds of neighbors.

### Nature of the response

Subjects reported extreme irritation, anger, and disgust as primary emotional responses (see Table 3). Aggressive outbursts were seldom reported at psychiatric interviews; verbal aggression was common, but physical aggression was rare. Frequency was not assessed with a questionnaire.

**Table 3:** Emotional response and hyper focus to sounds in misophonia subjects.

Emotional response to sounds		N = 257 (%)	
**Irritation**		**241 (93.8)**	
Severe to extreme irritation		248 (93.3)	
**Anger**		**230 (89.5)**	
Severe to extreme aggressive feeling		195 (73.8)	
Urge to hurt the person		208 (79.1)	
**Disgust**		**165 (64.0)**	
Severe to extreme disgust		167 (63.3)	
**Other emotional response**		**37 (13.6)**	
Synonym Anger		14 (5.1)	
Synonym Disgust		2 (0.7)	
Sadness		16 (5.9)	
Physical reaction		7 (2.6)	
Anxiety		5 (1.8)	
Loss of control		4 (1.5)	
Despair		3 (1.1)	
Boredom		1(0.4)	
Alienation		1 (0.4)	
**Perceived loss of control**		**208 (79.1)**	
Severe to extreme powerlessness		234 (88.9)	
**Hyper focus on sounds**		**N = 263 (%)**	
**Hyper focus**		**259 (98.1)**	
Severe to extreme hyper focus		241 (91.3)	
Seldom to never able to deviate attention		226 (85.6)	

None of the subjects reported anxiety as a primary response, neither at psychiatric examination nor with questionnaires. Only five subjects (1%) reported secondary anxiety, following anger or disgust. The majority of the sample reported anticipatory anxiety, which was mild and related to thinking of future misophonic situations. In clinical interviews, all subjects reported confrontations with triggers as stressful events. Subjects worried about misophonic triggers and their capacity to cope. A perceived loss of control was seen in 81% of the subjects and 90% reported severe to extreme powerlessness. In clinical interviews, most subjects expressed shame or guilt. Anticipatory anxiety and preoccupation with misophonic triggers appeared simultaneously. Two different scales showed 86 to 91% experienced serious preoccupation.

Most used coping was turning on music (99%) and walking away (99%). Making noise or making noise in the same rhythm, e.g., chewing or typing, was also used frequently, 86% and 77%, respectively. Finally, 86% described using earplugs; the majority of these subjects used them in the last week (73%).

In general, subjects reported to spend a lot of time actively avoiding triggers: 24% 0 to 1 hour each day, 24% 1 to 3 hours, 32% 3 to 8 hours, and 9% avoided over 8 hours each day.

### General somatic

76% of the sample reported no diagnosis on Axis III, 20% reported one diagnosis, and 4% had multiple diagnoses. Most common diagnoses were: migraine, irritable bowel syndrome, asthma, and back pain. During physical examination, a primary neurological disorder was never determined, but a mild somatic comorbid disorder was regularly found (e.g., hypertension). Approximately 1% of blood tests results were abnormal (e.g., decreased Hb levels, thyroid abnormalities or increased liver functions).

### Audiology

Of the total sample, four subjects (0.7%) were previously diagnosed with hyperacusis only, ten subjects (1.7%) with tinnitus only, and one patient with both. Twenty subjects reported hearing loss or other hearing problems.

The subgroup performing an audiogram consisted of 109 subjects (69% female) with a mean age of 36.70 years (*SD* = 12.08). 106 subjects had bilateral normal hearing (PTA < = 25 dB HL). The remaining three subjects had a unilateral hearing loss: one slight conductive hearing loss (25–40 dB HL), one moderate conductive hearing loss (40–60 dB HL), and one profound sensorineural hearing loss (80+ dB HL). This implies all participants had at least one ear with normal hearing.

### Severity

Subjects had moderate to moderate-severe symptoms according to the A-MISO-S and AMISOS-R. No significant sex differences were found (respectively *p* = .44 and *p* = .29). Subjects had mild symptoms on the HAS and HDRS and a high score on the SCL-90-R. Quality of life varied from a low satisfaction on the MANSA, to some impairment in day-to-day life on the SDS, particularly with family relations[40], to a slightly lower perceived quality of life on the WHOQoL-BREF[41]. Subjects all described to have made adjustments to their day-to-day life, such as avoiding public transport. Many subjects lost work or relationships because of misophonia. Rarely, subjects were desperate for help or expressed suicidal ideations in the clinical interviews.

### Personality profile

Subjects scored average on the AQ and ISS. Subjects scored low on the DS-R and on the DPSS-R[42]. There was a significant positive correlation between the DPSS-R and DS-R (r = .545, n = 442, *p* < .000). Further, subjects scored high on the CPQ (healthy controls in an unpublished AMC study have *M* = 23.82). A total of 97% had a score over 25, indicating clinical perfectionism. Subjects scored slightly higher on the FMPS (healthy controls in an unpublished AMC study have *M* = 92.70). A total of 66% had a score over 22 on the scale ‘Concern over mistakes’, indicating clinical perfectionism (cut off suggested by Egan & Hine[43]). There was no significant correlation between the CPQ and FMPS (r = .036, *p* = .571).

The NEO-PI-R sub-sample showed no sex differences and subjects scored above average (stanine 7) only on Neuroticism, with the facet Angry hostility (stanine 7). All characteristics are shown in Table 4.

**Table 4:** Characteristics of misophonia subjects.

Misophonia questionnaires	N	Mean (SD)
**AMISOS-R**	**258**	**29.78 (6.46)**
Female	183	30.00 (6.79)
Male	75	29.04 (5.76)
**A-MISO-S**	**253**	**14.02 (3.43)**
Female	175	14.00 (3.43)
Male	78	14.05 (3.43)
**General psychopathology**	**N**	**Mean (SD)**
**SCL-90-R**	**454**	**163.35 (53.17)**
**HAS**	**495**	**14.51 (9.54)**
**HDRS**	**436**	**10.97 (6.58)**
**GAF**	**516**	**68.05 (10.04)**
**Quality of life**	**N**	**Mean (SD)**
**MANSA**	**220**	**3.58 (0.73)**
**SDS total**	**98**	**17.79 (5.42)**
Work		5.33 (2.54)
Social		5.63 (2.22)
Family		6.79 (2.19)
**WHOQoL-BREF**	**102**	
Physical health		14.94 (2.42)
Psychological health		13.20 (2.12)
Social relationships		14.38 (2.47)
Environment General (1&2)		16.28 (1.84) 7.55 (1.44)
**Personality profile**	**N**	**Mean (SD)**
**AQ**	**109**	**19.25 (7.62)**
**IIS**	**221**	**76.90 (24.58)**
**CPQ**	**268**	**31.48 (8.99)**
**FMPS**	**261**	**94.67 (20.81)**
**DS-R**	**478**	**39.77 (13.38)**
**DPSS-R**	**464**	**23.35 (10.37)**
**NEO-PI-R**	**49**	
Neuroticism		152.7 (23.1)
Extraversion		148.3 (18.9)
Openness		156.7 (17.1)
Agreeableness		166.0 (17.4)
Consciousness		164.4 (19.2)

Using a standard multiple regression, perfectionism (CPQ: *p* = .487; FMPS: *p* = .651), autism traits (AQ: *p* = .270), and disgust sensitivity (DS-R: *p* = .628; DPSS-R: *p* = .961) showed no significant relation to the severity of the misophonia symptoms, measured by the AMISOS-R.

Finally, non-parametric correlation (Spearman’s rho) was used to determine whether misophonia symptoms (A-MISO-S) correlated with a decreased quality of life (MANSA). The more severe the misophonia symptoms, the lower the satisfaction with quality of life, r*s* (184) = -.34 *p* = < .001.

## Discussion

This is the largest qualitative and quantitative description of a sample of misophonia subjects so far (*N* = 575). Our study demonstrates that clinical examination from a medical-psychiatric perspective is invaluable for diagnosing misophonia, as one out of four referred subjects does not suffer from misophonia. Risk of misdiagnosis is high, because misophonia-like symptoms could be explained by comorbid conditions such as OCPD traits, mood disorders, AD(H)D, and ASC. From a somatic perspective, our misophonia subjects do not have specific somatic comorbid disorders. Furthermore, they have normal hearing, which is in contrast to hearing in tinnitus[44]. Prevalence of hearing loss found in our population is even less than expected based on the prevalence of disabling hearing loss in normal population for the Netherlands (PTA > 40 dB HL in better ear around 5% for adult population[45]). From a psychological perspective, misophonia can be seen as an independent construct. No association was found between misophonia symptoms and ASC, disgust sensitivity, or clinical perfectionism. Clinical perfectionism, however, was seen in 66 to 97% of the subjects. Severity of misophonia symptoms is negatively correlated with quality of life. Family relations especially suffer, but the influence on working life remains limited with only 5% on sick leave.

Overall prevalence rate of comorbid DSM-IV Axis I disorders is similar to general population in the Netherlands, except for mood disorders (twice as prevalent[46]), AD(H)D (two-and-a-half times more prevalent[46]) and the ASC (threefold the prevalence[47]). Some studies[6, 48] suggest an association with affective disorders, particularly post-traumatic stress disorder. The preliminary results of a new sample study using psychiatric evaluation[9] even showed a prevalence of 15%. However, prevalence of PTSD in our sample is not higher. The prevalence rate of comorbid DSM-IV Axis II disorders is mildly higher and corresponds with findings of Rouw & Erfanian[6]. We found OCPD traits in one out of four subjects. Regardless, the exact prevalence of personality traits in community samples is unknown, we consider a 26% prevalence of OCPD traits high. 52% of the 2013 AMC sample[2] had a comorbid OCPD. This difference can be explained by a smaller sample size and selection bias in this previous sample.

Development and severity of misophonia symptoms in this large sample are consistent with findings in the 2013 AMC sample[2]. In our sample, however, a larger percentage is female. Specifically, age of onset, course, severity of symptoms, and a positive family history[49, 6, 7] supports misophonia as a distinct disorder[50]. Findings from psychiatric, medical, and psychological assessments substantiate this conclusion. Our findings result in a revision of the 2013 criteria, which are illustrated in the next paragraph and marked in Table 5. We emphasize that to be diagnosed with misophonia, all criteria should be met. As in all psychiatric disorders a subclinical group probably exists, in most cases lacking criterion E-R.

**Table 5:** Amsterdam UMC 2020 revised diagnostic criteria for misophonia.

Amsterdam UMC 2020 revised criteria for misophonia
A-R. Preoccupationᵃ with a specific auditory, visual or sensory cue^c^, which is predominantly induced by another personᵈ. It is required that oral or nasal sounds are a trigger.ᵇ
B-R. Cues evoke intense feelings of irritation, anger and/or disgust of which the individual recognizes it is excessive, unreasonable or out of proportion to the circumstances.
C-R. Since emotions trigger an impulsive aversive physical reaction, the individual experiences a profound sense of loss of self-control with rare but potentially aggressive outbursts.
D-R. The individual actively avoids situations in which triggers occur or endures triggers with intense discomfort, irritation, anger or disgust.
E-R. The irritation, anger, disgust or avoidance causes significant distress and/or significant interference in the individual’s day-to-day life. For example, it is impossible to eat together, work in an open office space or live together.ᵉ
F-R. The irritation, anger, disgust and avoidance are not better explained by another disorder, such as an Autism Spectrum Condition (e.g. a general hypersensitivity or hyper arousal to all sensory stimuli)ᶠ or Attention Deficit Hyperactivity Disorder (e.g. attention problems with high distractibility in general)ᶠ.

### Misophonia triggers

Our detailed investigation of triggers leads to new conclusions. Approximately all subjects in our sample report eating sounds as a trigger (96%) and the majority reports nasal or breathing sounds as a trigger (85%). Combined, all subjects report either oral or nasal sounds as a trigger. Therefore, we propose other triggers can be a part of the condition, but an emotional reaction to oral or nasal sounds is requiredᵇ. Visual triggers, like scratching, and non-human triggers, like animal sounds or air-conditioning sounds, were occasionally described[51, 52]. We indeed found evidence for non-auditory triggers in 78% of our sample, but auditory triggers are primary triggers^c^. If combined, these triggers cause a more intense emotional reaction. In a mass experiment which was performed among the general population, adding a corresponding image to a disgusting sound had no effect[53]. This interaction effect is probably typical for misophonia subjects.

Ambient sounds are most often reported amongst other misophonia triggersᵈ (see S2 Table). If subjects are bothered only by ambient sounds (e.g., sounds of neighbors), misophonia should not be diagnosed, even though subjects describe a similar response. Over 8% of the Dutch population reported serious nuisance by sounds of neighbors in the last year and 29% reported mild or moderate nuisance. A much higher percentage is bothered by various traffic sounds[54]. In these cases, symptoms can be seen as a more general disturbance of sounds, such as noise sensitivity or sensory over-responsivity, which also occurs in a normal population[55]. We used typical examples of avoidance from our psychiatric assessmentsᵉ.

### Nature of the response

As in other samples and case studies, we show misophonia is associated with considerable non-expressed aggression, but physical aggressive outbursts are rare[3]. Subjects with a comorbid affective instability, due to for instance comorbid BPD, sometimes reported aggressive outbursts in our clinical interviews. These outbursts were mild in comparison to the internal aggressive thoughts all misophonia subjects described. Anxiety is also frequently described as a response to misophonia triggers[56, 57, 5, 8]. In our sample, subjects do not report anxiety as a prompt reaction to a trigger, but experience anticipatory anxiety and physical stress. Possibly this anticipatory anxiety is elsewhere mistaken for anxiety as a primary response. This emphasizes the value of a thorough psychiatric evaluation once more. We found all subjects consider their reaction to be out of proportion. Interestingly, only one case study describes a patient who perceived her reaction to sounds as inappropriate[58]. Subjects with a primary OCPD or ASC often do not consider their reaction to be out of proportion, and OCPD or ASC are possible differential diagnosesᶠ.

Subjects often stated the emotional response to be far more intense towards loved ones inducing misophonia triggers. Context also influenced the emotional response; when misophonia trigger sounds were made by toddlers, mentally disabled adults, or elderly with dementia, an emotional response seldom occurred.

We consider preoccupation an additional core symptom of misophonia, since approximately all subjects in our sample report hyper focusᵃ. Hyper focus was also reported in 82% of the sample of Edelstein et al[49] and an intervention targeting hyper focus has a clear effect on misophonia symptoms[59].

### Limitations

This is the first large sample study of misophonia subjects in which we not only explored the dynamics of misophonia symptoms, but also examined the impact of symptoms on quality of life. However, this research also has its limitations. First, the Amsterdam UMC is the only center in the Netherlands with a specific treatment for misophonia, which could lead to a selection bias. Furthermore, since no other criteria were available, AMC 2013 diagnostic criteria were used for selection of subjects, possibly leading to confirmation bias. However, we believe we limited confirmation bias, because we examined all patients who were referred with misophonia-like symptoms; using questionnaires with a broad scope, and we investigated alternative symptoms, e.g., anxiety, in our psychiatric evaluations. Finally, types or versions of questionnaires administered changed over time in this sample. Some of the questionnaires administered are not yet validated in Dutch translation or lack a norm group, but were the best available. Also, the A-MISO-S is not psychometrically validated and the AMISOS-R is in the process of validation.

### Conclusions

In conclusion, this analysis of a large sample confirms that misophonia is a distinct psychiatric disorder characterized by an intense emotional reaction of irritation, anger, and often disgust elicited by specific auditory, visual or sensory triggers predominantly induced by another person, resulting in preoccupation and avoidance. We suggest future studies to use the revised Amsterdam UMC proposed criteria and to conduct international multi-center studies. A multi-disciplinary approach, especially including psychiatry, audiology, and psychology, would be preferable. International confirmation of the Amsterdam UMC revised criteria is needed before next steps in research can be taken. Future research should also include more treatment studies (e.g., a RCT of CBT or a new intervention) and validation of misophonia questionnaires.

## Supporting information

S1 Fig Flowchart search May, 2018. (DOCX)

S1 Table Results search May, 2018. (DOCX)

S2 Table Overview questionnaires. (DOCX)

S3 Table Triggers for misophonia subjects. (DOCX)

S1 Appendix Misophonia screening list. (PDF)

S2 Appendix Misophonia Sound List (MSL). (PDF)

S3 Appendix Revised Amsterdam Misophonia Scale (AMISOS-R). (PDF)
