# Supplementary material for: Misophonia: Phenomenology, comorbidity and demographics in a large sample
Source: PLoS One. 2020 Apr 15;15(4):e0231390. doi: 10.1371/journal.pone.0231390 (PMC7159231; doi:10.1371/journal.pone.0231390)
Supplement: S1 Fig — (DOCX) [file pone.0231390.s001.docx]

**S.1 Fig. Flowchart search May, 2018*.***

Embase 52

Pubmed 44 **121**

Psychinfo 25

**40** full text articles assessed

**56** abstracts screened

**16** full text articles excluded

8 reviews, 3 commentaries

3 experimental studies

1 treatment study

1 sample study

**63** doubles removed

**2** articles not found

**24** articles included

13 case studies, 5 experimental studies, 5 sample studies, 1 treatment study
