# Supplementary material for: Misophonia: Phenomenology, comorbidity and demographics in a large sample
Source: PLoS One. 2020 Apr 15;15(4):e0231390. doi: 10.1371/journal.pone.0231390 (PMC7159231; doi:10.1371/journal.pone.0231390)
Supplement: S1 Table — (DOCX) [file pone.0231390.s002.docx]

**S1 Table. Results search May, 2018.**

|  | ***Study authors*** | ***Study type*** | ***Study population*** | ***Diagnosis based on*** |
| --- | --- | --- | --- | --- |
| **1** | **Bernstein et al., 2013** | Case study | 1 misophonia pt | Clinician |
| **2** | **Bruxner, 2016** | Review and case vignette | 1 misophonia pt | Clinician |
| **3** | **Dozier, 2015** | Case study | 1 misophonia pt | Clinician, questionnaires (MAQ, MCR, MTS) |
| **4** | **Edelstein et al., 2013** | Experimental study | 11 misophonia pts, 5 controls | Self-diagnosed |
| **5** | **Eijsker et al., 2017** | Experimental study | 21 misophonia pts, 23 controls | Clinician, questionnaire (A-MISO-S) |
| **6** | **Ferreira et al., 2013** | Case study | 3 misophonia pts | Clinician |
| **7** | **Johnson et al., 2013** | Case study | 4 misophonia pts | Clinician |
| **8** | **Kamody et al., 2017** | Case study | 1 misophonia pt | Clinician, questionnaires (A-MISO-S, MAQ) |
| **9** | **Kluckow et al., 2014** | Case study | 3 misophonia pts | Clinician, questionnaires (A-MISO-S, MAS-1) |
| **10** | **Kumar et al., 2017** | Experimental study | 20 misophonia pts, 22 controls | Questionnaire (unclear: Kumar 2014) |
| **11** | **McGuire et al., 2015** | Case study | 2 misophonia pts | Questionnaires (MQ and MSS) |
| **12** | **McKay et al., 2015** | Sample study: profile analysis | 121 misophonia pts, 507 controls | Questionnaires (MQ) |
| **13** | **Neal et al., 2013** | Case study | 1 misophonia pt | Clinician |
| **14** | **Reid et al., 2016** | Case study | 1 misophonia pt | Clinician, Questionnaire (A-MISO-S) |
| **15** | **Rouw et al., 2017** | Sample study | 301 misophonia pts | Self-diagnosed Questionnaires (MAS, MPRS, A-MISO-S) |
| **16** | **Sanchez et al., 2017** | Sample study | 12 misophonia pts | Self-diagnosed |
| **17** | **Schröder et al., 2013** | Sample study | 42 misophonia pts | Clinician, Questionnaire (A-MISO-S) |
| **18** | **Schröder et al., 2014** | Experimental study | 20 misophonia pts, 14 controls | Clinician, Questionnaire (A-MISO-S) |
| **19** | **Schröder et al., 2015** | Experimental study | 10 misophonia pts, 7 controls | Clinician, Questionnaire (A-MISO-S) |
| **20** | **Schröder et al., 2017** | Treatment study | 90 misophonia pts | Clinician, Questionnaire (A-MISO-S) |
| **21** | **Veale, 2006** | Case study | 1 possible misophonia pt | Clinician |
| **22** | **Webber et al., 2014** | Case study | 1 misophonia pt | Clinician |
| **23** | **Wu et al., 2014** | Sample study | 483 students of which 20%: 96 misophonia pts | Questionnaire (MQ) |
| **24** | **Zhou et al., 2017** | Sample study | 415 students of which 6%: 25 misophonia pts | Questionnaires (MQ, SDS-M) |

*MAQ = Misophonia Assessment Questionnaire, MCR = Misophonia Coping Responses, MTS = Misophonia Trigger Severity scale, A-MISO-S = Amsterdam Misophonia Scale, MQ = Misophonia Questionnaire, MSS = Misophonia Severity Scale, MAS-1 = Misophonia Activation Scale, MPRS = Misophonia Physical Sensation Scale, SDS-M = Sheehan Disability Scale for Misophonia*
