# Supplementary material for: Misophonia: Phenomenology, comorbidity and demographics in a large sample
Source: PLoS One. 2020 Apr 15;15(4):e0231390. doi: 10.1371/journal.pone.0231390 (PMC7159231; doi:10.1371/journal.pone.0231390)
Supplement: S2 Table — (DOCX) [file pone.0231390.s003.docx]

**S.2 Table. Overview questionnaires.**

| **Questionnaire** | **Interpretation** | ***N* total** | ***N* completed (%)** |
| --- | --- | --- | --- |
| **Misophonia screening list**  (see S1 Appendix) | range 0-56, higher scores (>20) indicating misophonia | 275 | 264 (96) |
| **A-MISO-S***  (Schröder et al., 2013) | range from 0-24, higher scores indicating more severe misophonia. 0–4 subclinical misophonic symptoms, 5–9 mild, 10–14 moderate, 15–19 severe, 20–24 extreme | 275 | 253 (92) |
| **AMISOS-R***  (see S3 Appendix) | range 0-40, higher scores indicating more severe misophonia. 0–10 subclinical misophonic symptoms, 11–20 mild, 21–30 moderate severe, 31–40 severe to extreme | 300 | 259 (86) |
| **MSL**  (see S2 Appendix) | range 0-4, higher scores indicating more provoked anger | 275 | 135 (49) |
| **HDRS***  (De Jonge, 1994; Hamilton, 1960) | range 0-54, higher scores indicating more symptoms of depression | 575 | 436 (76) |
| **HAS***  (Maier et al., 1988; Hamilton, 1959) | range 0-56, higher scores indicating more anxiety | 575 | 495 (86) |
| **SCL-90-R***  (SCL-90-R; Arrindell & Ettema, 1986; Derogatis et al., 1973) | range 90-450, higher scores indicating more general psychopathology | 575 | 454 (71) |
| **MANSA**  (Nieuwenhuizen et al., 2017; Priebe et al., 1999) | range 0-7, higher scores indicating greater life satisfaction | 275 | 220 (80) |
| **SDS**  (Sheehan, 1983) | range 0-10, higher scores indicating more impairment | 115 | 98 (85) |
| **WHOQoL-BREF**  (Trompenaars et al., 20015; WHOQoL Group, 1998) | range per domain 4-20, range general health 1-10, higher scores indicating a higher perceived quality of life | 115 | 102 (89) |
| **NEO-PI-R ◊**  (Miller et al., 2008; Costa et al., 1992) | with scores on five subscales neuroticism, extraversion, openness, agreeableness and consciousness | 56 | 49 (88) |
| **AQ**  (Hoekstra et al., 2008; Baron-Cohen et al., 2001) | range from 0-50, higher scores indicating more evidence for Autism Spectrum Conditions | 115 | 109 (95) |
| **ISS**  (Van Dam et al., 2000) | range 35-175, higher scores indicating more social anxiety | 275 | 212 (77) |
| **CPQ**  (CPQ; Shafran et al., 2002) | range 12-48, with higher scores indicating more perfectionism | 300 | 268 (89) |
| **FMPS**  (Frost et al., 1990) | range 35-175, higher scores indicating more perfectionism | 300 | 261 (87) |
| **DPSS-R***  (Van Overveld et al., 2006; Cavanagh et al., 2000) | range 0-64, higher scores indicating more propensity and sensitivity to disgust | 575 | 464 (81) |
| **DS-R***  (Van Overveld et al., 2011; Haidt et al., 1994) | range 0-100, higher scores indicating more disgust sensitivity | 575 | 478 (83) |

***** seven questionnaires were presented to all subjects

**◊** questionnaire presented to a random sample
