# Supplementary material for: Misophonia: Phenomenology, comorbidity and demographics in a large sample
Source: PLoS One. 2020 Apr 15;15(4):e0231390. doi: 10.1371/journal.pone.0231390 (PMC7159231; doi:10.1371/journal.pone.0231390)
Supplement: S3 Table — (DOCX) [file pone.0231390.s004.docx]

**S3 Table. Triggers for misophonia subjects on MSL.**

| **Reported triggers** | ***Total N*** | **Mean (*SD*)** |
| --- | --- | --- |
| **Eating sounds** | **424** |  |
| Smacking |  | 3.27 (0.8) |
| Biting an apple |  | 2.50 (1.4) |
| Cracking of crisps |  | 2.92 (1.2) |
| Swallowing |  | 2.31 (1.3) |
| Slurping |  | 2.68 (1.1) |
| Gulp drinking sounds |  | 2.44 (1.3) |
| Chewing gum |  | 2.89 (1.1) |
| **Nasal and breathing sounds** | **425** |  |
| Breathing |  | 2.26 (1.3) |
| Sniffling and nostril sounds |  | 2.52 (1.3) |
| Sneezing |  | 0.89 (1.2) |
| Snoring |  | 2.62 (1.2) |
| **Mouth and throat sounds** | **418** |  |
| Yawning |  | 1.08 (1.3) |
| Clearing the throat |  | 1.66 (1.3) |
| Coughing |  | 1.57 (1.3) |
| Whistling |  | 1.41 (1.4) |
| Kissing |  | 1.23 (1.3) |
| **Certain vocal sounds** | **257** |  |
| **Sounds of repetitive tapping** | **420** |  |
| Typing and clicking |  | 1.69 (1.5) |
| Pen clicking |  | 1.94 (1.5) |
| Nail tapping |  | 1.79 (1.4) |
| Nail clipping |  | 2.02 (1.4) |
| Cutlery and tableware |  | 1.39 (1.3) |
| **Rustling sounds** | **420** |  |
| Rustling plastic bag |  | 1.26 (1.3) |
| Turning pages |  | 0.82 (1.1) |
| **Ambient sounds** | **420** |  |
| Phone calls in public |  | 1.13 (1.2) |
| Neighbors speaking |  | 1.63 (1.4) |
| Music neighbors |  | 2.01 (1.5) |
| Devices (e.g., washer, clock, hoover) |  | 1.20 (1.3) |
| Pets (eg spinning cat) |  | 0.97 (1.3) |
| **Repetitive movements** | **166** |  |
| Scratching |  | 0.76 (1.1) |
| Rocking legs |  | 1.92 (1.4) |
| Fiddling fingers |  | 1.74 (1.5) |
