# Supplementary material for: Misophonia: Phenomenology, comorbidity and demographics in a large sample
Source: PLoS One. 2020 Apr 15;15(4):e0231390. doi: 10.1371/journal.pone.0231390 (PMC7159231; doi:10.1371/journal.pone.0231390)
Supplement: S1 Appendix — (PDF) [file pone.0231390.s005.pdf]

Misophonia Screening List

Name:	__________
Date of birth:	___-___-___
Date of session:	___-___-___

Choose the answer that is most applicable for you.

1. I am focused on sounds people make	**Very much disagree**<br><br>**0**	**1**	**2**	**3**	**Very much agree**<br><br>**4**
2. If people make sounds, I instantly walk away	**Very much disagree**<br><br>**0**	**1**	**2**	**3**	**Very much agree**<br><br>**4**
3. I made arrangements to my life to minimize hearing sounds	**Very much disagree**<br><br>**0**	**1**	**2**	**3**	**Very much agree**<br><br>**4**
4. I want to hurt the person making sounds	**Very much disagree**<br><br>**0**	**1**	**2**	**3**	**Very much agree**<br><br>**4**
5. If I enter a room in which my loved ones are eating, I feel aggression	**Very much disagree**<br><br>**0**	**1**	**2**	**3**	**Very much agree**<br><br>**4**
6. If I enter a room in which my loved ones are eating, I feel disgust	**Very much disagree**<br><br>**0**	**1**	**2**	**3**	**Very much agree**<br><br>**4**

7. I feel annoyed by sounds all day	**Very much disagree**<br><br>**0**	**1**	**2**	**3**	**Very much agree**<br><br>**4**
8. My quality of life is greatly decreased due to sounds of others	**Very much disagree**<br><br>**0**	**1**	**2**	**3**	**Very much agree**<br><br>**4**
9. I would be able to relax more if I did not hear any sounds	**Very much disagree**<br><br>**0**	**1**	**2**	**3**	**Very much agree**<br><br>**4**
10. I suffer from sounds other people produce	**Very much disagree**<br><br>**0**	**1**	**2**	**3**	**Very much agree**<br><br>**4**
11. My relationships are limited by sounds others make	**Very much disagree**<br><br>**0**	**1**	**2**	**3**	**Very much agree**<br><br>**4**
12. I feel interference in my day-to-day life by the sounds of others	**Very much disagree**<br><br>**0**	**1**	**2**	**3**	**Very much agree**<br><br>**4**
13. After hearing a sound, I experience a sense of loss of self-control	**Very much disagree**<br><br>**0**	**1**	**2**	**3**	**Very much agree**<br><br>**4**
14. I feel misunderstood when I suffer from sounds	**Very much disagree**<br><br>**0**	**1**	**2**	**3**	**Very much agree**<br><br>**4**
**Total score:**					
