# Supplementary material for: Misophonia: Phenomenology, comorbidity and demographics in a large sample
Source: PLoS One. 2020 Apr 15;15(4):e0231390. doi: 10.1371/journal.pone.0231390 (PMC7159231; doi:10.1371/journal.pone.0231390)
Supplement: S2 Appendix — (PDF) [file pone.0231390.s006.pdf]

Misophonia Sound List

Name:	___________
Date of birth:	___-___-___
Date of session:	___-___-___

Category A

never	past	last week	Sounds	Degree of irritation/disgust/anger<br>0 = no<br>1 = a little (e.g., irritation)<br>2 = quite<br>3 = a lot (e.g., anger)<br>4 = extreme (e.g., rage)
			Smacking	0 – 1 – 2 – 3 – 4
			Biting an apple	0 – 1 – 2 – 3 – 4
			Crunching of crisps	0 – 1 – 2 – 3 – 4
			Swallowing	0 – 1 – 2 – 3 – 4
			Slurping	0 – 1 – 2 – 3 – 4
			Gulping	0 – 1 – 2 – 3 – 4
			Breathing	0 – 1 – 2 – 3 – 4
			Sniffling and nostril sounds	0 – 1 – 2 – 3 – 4
			Yawning	0 – 1 – 2 – 3 – 4
			Clearing the throat	0 – 1 – 2 – 3 – 4
			Footsteps or high heels on the floor	0 – 1 – 2 – 3 – 4
			Chewing gum	0 – 1 – 2 – 3 – 4
			Coughing	0 – 1 – 2 – 3 – 4
			Sneezing	0 – 1 – 2 – 3 – 4
			Whistling	0 – 1 – 2 – 3 – 4
			Typing and clicking	0 – 1 – 2 – 3 – 4

			Pen clicking	0 – 1 – 2 – 3 – 4
			Nail tapping	0 – 1 – 2 – 3 – 4
			Nail clipping	0 – 1 – 2 – 3 – 4
			Cutlery and tableware	0 – 1 – 2 – 3 – 4
			Turning pages	0 – 1 – 2 – 3 – 4
			Scratching the head	0 – 1 – 2 – 3 – 4
			Rustling plastic bag	0 – 1 – 2 – 3 – 4
**Total score A:**				

Category B

**never**	**past**	**last week**	**Other sounds**	**Degree of irritation/disgust/anger**<br>0 = no<br>1 = a little (e.g., irritation)<br>2 = quite<br>3 = a lot (e.g., anger)<br>4 = extreme (e.g., rage)
			Snoring	0 – 1 – 2 – 3 – 4
			Phone calls in public	0 – 1 – 2 – 3 – 4
			Kissing	0 – 1 – 2 – 3 – 4
			Neighbors speaking	0 – 1 – 2 – 3 – 4
			Music from neighbors	0 – 1 – 2 – 3 – 4
**Total score B:**				

Category C

**never**	**past**	**last week**	**Non-human sounds**	**Degree of irritation/disgust/anger**<br>0 = no<br>1 = a little (e.g., irritation)<br>2 = quite<br>3 = a lot (e.g., anger)<br>4 = extreme (e.g., rage)
			Devices (e.g., washer, clock, hoover)	0 – 1 – 2 – 3 – 4

			Pets (e.g., dog licking, cat purring, rooster crowing)	0 – 1 – 2 – 3 – 4
**Total score C:**				

Category D

**never**	**past**	**last week**	**Visual triggers**	**Degree of irritation/disgust/anger**<br>0 = no<br>1 = a little (e.g., irritation)<br>2 = quite<br>3 = a lot (e.g., anger)<br>4 = extreme (e.g., rage)
			Twitching legs	0 – 1 – 2 – 3 – 4
			Fiddling fingers	0 – 1 – 2 – 3 – 4
**Total score D:**				

Category E

**never**	**past**	**last week**	**Avoidance**	**Degree of avoidance**<br>0 = no<br>1 = a little<br>2 = quite<br>3 = a lot<br>4 = extreme
			Putting on music (speakers or headphone)	0 – 1 – 2 – 3 – 4
			Making noise	0 – 1 – 2 – 3 – 4
			Making noise in the same rhythm (e.g., 'chewing along')	0 – 1 – 2 – 3 – 4
			Wearing earplugs	0 – 1 – 2 – 3 – 4
			Walking away	0 – 1 – 2 – 3 – 4
**Total score E:**				
