# Supplementary material for: Misophonia: Phenomenology, comorbidity and demographics in a large sample
Source: PLoS One. 2020 Apr 15;15(4):e0231390. doi: 10.1371/journal.pone.0231390 (PMC7159231; doi:10.1371/journal.pone.0231390)
Supplement: S3 Appendix — (PDF) [file pone.0231390.s007.pdf]

Amsterdam Misophonia Scale - Revised

(AMISOS-R)

Name:	___________
Date of birth:	___-___-___
Date of session:	___-___-___

In comparison to others I am sensitive to:

(multiple answers possible)

- o Eating sounds (e.g. chewing, smacking, slurping, swallowing)
- o Nasal sounds (e.g. sniffing, breathing in, breathing out)
- o Throat sounds (e.g. harrumphing, coughing)
- o Specific sounds (e.g. ‘k’ sound)
- o Repeating clicking sounds (e.g. nails on a blackboard, pen clicking)
- o Crinkling sounds (e.g. paper, plastic)
- o Ambient noises (e.g. clock ticking noise or similar devices)

Which emotions are evoked by listening to those sounds?
(multiple answers possible)

- o Irritation
- o Anger
- o Disgust
- o Other:

Review your experience from hearing your misophonia sounds *in the last 3 days*. Read instead of “sounds” your most disturbing misophonia sounds and instead of “emotion” your typical emotion. Choose the answer that is most applicable for you.

1. How many time do you spend a day (thinking about) these sounds?	**0 hour**<br><br>**0**	**< 1 hour**<br><br>**1**	**1-3 hour**<br><br>**2**	**3-8 hour**<br><br>**3**	**>8 hour**<br><br>**4**
2. To what extent do you focus on these sounds?	**not**<br><br>**0**	**mild**<br><br>**1**	**moderate**<br><br>**2**	**severe**<br><br>**3**	**extreme**<br><br>**4**
3. To what extent do you experience impairment due to these sounds?	**not**<br><br>**0**	**mild**<br><br>**1**	**moderate**<br><br>**2**	**severe**<br><br>**3**	**extreme**<br><br>**4**
4. How intense is your feeling of irritability/anger when you hear these sounds?	**not**<br><br>**0**	**mild**<br><br>**1**	**moderate**<br><br>**2**	**severe**<br><br>**3**	**extreme**<br><br>**4**
5. To what extent do you feel helpless against these sounds?	**not**<br><br>**0**	**mild**<br><br>**1**	**moderate**<br><br>**2**	**severe**<br><br>**3**	**extreme**<br><br>**4**
6. To what extent are you suffering from these sounds?	**not**<br><br>**0**	**mild**<br><br>**1**	**moderate**<br><br>**2**	**severe**<br><br>**3**	**extreme**<br><br>**4**
7. To what extent are you suffering from the avoidance of these sounds?	**not**<br><br>**0**	**mild**<br><br>**1**	**moderate**<br><br>**2**	**severe**<br><br>**3**	**extreme**<br><br>**4**
8. To what extent are the sounds limiting your life (work, household etc.)?	**not**<br><br>**0**	**mild**<br><br>**1**	**moderate**<br><br>**2**	**severe**<br><br>**3**	**extreme**<br><br>**4**
9. To what extent are you avoiding specific places or situations because of the sounds?	**not**<br><br>**0**	**mild**<br><br>**1**	**moderate**<br><br>**2**	**severe**<br><br>**3**	**extreme**<br><br>**4**
10. To what extent can you shift your attention when you are hearing these sounds?	**always**<br><br>**0**	**usually (75%)**<br><br>**1**	**sometimes (50%)**<br><br>**2**	**seldom (25%)**<br><br>**3**	**never**<br><br>**4**
**Total score:**					
